# Medical and Dietary Uses of N-Acetylcysteine

**DOI:** 10.3390/antiox8050111

**Published:** 2019-04-28

**Authors:** Špela Šalamon, Barbara Kramar, Tinkara Pirc Marolt, Borut Poljšak, Irina Milisav

**Affiliations:** 1 Center for human molecular genetics and pharmacogenomics, Faculty of Medicine, University of Maribor, SI-2000 Maribor, Slovenia; spela.salamon@gmail.com; 2 Institute of Pathophysiology, Faculty of Medicine, University of Ljubljana, Zaloška 4, SI-1000 Ljubljana, Slovenia; barbara.kramar@mf.uni-lj.si (B.K.); tinkara.pircmarolt@mf.uni-lj.si (T.P.M.); 3 University of Ljubljana, Faculty of Health Sciences, Laboratory of Oxidative Stress Research, Zdravstvena pot 5, SI-1000 Ljubljana, Slovenia; borut.poljsak@zf.uni-lj.si

**Keywords:** N-acetylcysteine, acetylcysteine, pharmacology, physiology, dietary supplements, aging, sports

## Abstract

N-acetylcysteine (NAC), a plant antioxidant naturally found in onion, is a precursor to glutathione. It has been used as a drug since the 1960s and is listed on the World Health Organization (WHO) Model List of Essential Medicines as an antidote in poisonings. There are numerous other uses or proposed uses in medicine that are still in preclinical and clinical investigations. NAC is also used in food supplements and cosmetics. Despite its abundant use, there are projections that the NAC global market will grow in the next five years; therefore, the purpose of this work is to provide a balanced view of further uses of NAC as a dietary supplement. Although NAC is considered a safe substance, the results among clinical trials are sometimes controversial or incomplete, like for many other antioxidants. More clinical trials are underway that will improve our understanding of NAC applicability.

## 1. Introduction

N-acetylcysteine (also known as N-acetyl-cysteine, NAC) is a precursor to the amino acid L-cysteine and consequently the antioxidant glutathione (GSH) [1]. It is most notably found in plants of the *Allium* species, especially in the onion (*Allium cepa*, 45 mg NAC/kg) [2,3]. The sulfhydryl group (–SH) within the NAC molecule directly scavenges reactive oxygen species (ROS) [4], modulates the redox state of the N-methyl-D-aspartate (NMDA) and α-amino-3-hydroxy-5-methyl-4-isoxazolepropionic acid (AMPA) receptors (neurotransmitter effect) [5], and inhibits the nuclear factor kappa-light-chain-enhancer of activated B cells (NF-κB) to modulate cytokine synthesis (anti/pro-inflammatory effect) [6]. Unlike GSH itself, NAC has better oral and topical bioavailability [7,8]. Even though it has been used for more than 50 years, there are still many controversies surrounding it as a medicine as well as a dietary supplement. Several review articles have focused on various medical uses of NAC, some more general [9] and others highly specific dealing with NAC use only in a particular condition such as hyperglycaemia-induced oxidative damage [10], liver diseases [11], and traumatic brain injury [12]. Other reviews have discussed the use of NAC as a dietary supplement in psychiatric conditions by itself [13] or in conjunction with other supplements [14,15]. However, we were unable to find a review of both medical and supplementary uses of NAC.

## 2. Molecular Mechanisms: Both Sides of the Redox Equation

GSH is the most abundant intracellular free thiol, and its decrease (and the increase in its redox couple oxidized glutathione, GSSG) has a crucial role in cell oxidative capacity [16]. It is required for stem cell function [17], and its depletion triggers cell death pathways [18]. Cellular oxidative stress is often seen as a GSH deficiency that is characteristic of many pathological conditions such as heart disease [19], diabetes [20], neurodegenerative disease [21], AIDS [22], as well as normal aging [23]. Though primarily seen as an antioxidant, NAC also has pro-oxidative effects. While NAC can scavenge several ROS (including HOCl, ONOO^−^, RO_2_^●^) [24] and hydroxyl radicals (OH^●^) [25], it has a poor ability for sequestering O_2_^●−^ [26] and hydrogen peroxide (H_2_O_2_) in vitro [27]. NAC can behave as an oxidant by undergoing auto-oxidation in high concentrations or in the presence of transition metals. NAC can reduce transitional metals and promote the formation of ROS via Fenton-like chemistry or the production of thiyl radical:

NAC(SH) + Fe^3+^ → NAC(S^●−^) + Fe^2+^ + H^+^ (1)

For example, by reducing ferric iron to its catalytic, active Fe^2+^ form, NAC enhances the production of hydroxyl radicals in the in vitro system with Fe(III)-citrate and H_2_O_2_ [28]. Dose-dependent oxidative damage to DNA was observed in the presence of NAC in the presence of the transition metal, copper [29]. Besides, the products of pro-oxidant reactions mediated by NAC are involved in altering the redox-sensitive NF-κB signal transduction pathway activation; mitogen activated protein kinase p38 (p38MAPK) and c-Jun N-terminal kinase (JNK) phosphorylation [30,31]. The antioxidant and pro-oxidant effects of NAC have been discussed in greater detail elsewhere [32].

## 3. Medical Use of NAC

NAC has been an established drug since the 1960s; it is on the World Health Organization’s List of 40 Essential Medicines [33] and is available as an inexpensive generic drug. It has been classically used in paracetamol overdose [34] and as a mucolytic [35], as well as to combat the toxicity of various substances that can cause generation of free radicals, such as carbon monoxide and x-ray contrasts [36]. The NAC products currently approved by Food and Drug Administration (FDA) are listed in Table 1. NAC is also used in the complementary treatment of neurological and neuropsychiatric disorders [5,35]. One death due to an anaphylactic reaction was described following an intravenous (IV) injection of 150 mg/kg of NAC in a 40 year old asthmatic woman in 2002. At comparable IV doses, vomiting was also reported in 11% of patients [37]. However, oral NAC seems to be associated with very few side effects and is considered to have an excellent safety profile [35]. One case of angioedema after oral NAC administration was described in 1997 [38]. Clinical studies have revealed benefits of NAC also in non-alcoholic steatohepatitis [39], arterial hypertension of diabetic etiology [40], chronic obstructive pulmonary disease (COPD) [41,42] and chronic bronchitis [43], substance abuse disorders [44], recurrent unexplained pregnancy loss [45], male infertility [46], polycystic ovary syndrome [47], diabetic retinopathy, age-related macular degeneration, and cataract and dry eye syndrome [4]. In total, 300 clinical studies (291 clinical trials) of NAC are listed in ClinicalTrials.gov [48] in April 2019 (Table 2). The most common disorders that were investigated by listed interventional trials with NAC (without the currently active studies) included renal disorders (48 trials) with an emphasis on radiocontrast nephropathy prevention, chronic kidney disease, and renoprotection during surgery; and neurological and psychiatric disorders (36 trials), leading with Parkinson’s disease, schizophrenia, bipolar, autistic, and behavioral disorders. Schizophrenia, for instance, has been linked to mitochondrial abnormalities, glutathione deficiency, and increased oxidative stress in the brain. Negative and general symptoms in schizophrenia may be reduced after 8–24 weeks of adjunctive treatment with NAC [49] in neuropsychiatric disorders and are discussed in greater detail in a recent review [50]. Addictive disorders (23 trials) are also a common target, with alcohol, tobacco, cocaine, cannabis, and other types of dependence. The NMDA receptors that NAC modulates may be involved in addiction [51], and at least three reviews discuss the use of NAC in addictive disorders [44,52,53] and emphasize the reduction of cravings for the substance in question. Among other commonly investigated uses of NAC were applications in gastrointestinal and pulmonary diseases. The majority of the 54 currently active interventional studies are investigating the role of NAC in addictive disorders, mental health, and neurodegenerative diseases, followed by cancer/cancer treatment side-effects, cardiovascular diseases, and surgery complications/trauma.

The suspended, terminated, or withdrawn studies listed in ClinicalTrals.gov are in Table 3. Termination reasons, such as no improvement and opposite results, are recorded in only 3 out of the 23 trials. Insufficient funds and insufficient recruitment are the major termination/ suspension/ withdrawal reason [48]. There are a few reports of the NAC study premature termination in the literature. High doses of NAC did not improve respiratory health in patients with COPD and chronic bronchitis; the study was prematurely terminated [54]. The decision was based on a potential safety issue, as it was reported that NAC and vitamin E, given orally, induced lung cancer in mice. This finding was reproduced in cell lines from human and mice lung tumors [55]. Additionally, there was no indication of improvement of COPD/chronic bronchitis in the 23 patients that received 1800 mg NAC twice daily for 8 weeks compared to the equal number of subjects receiving placebo [54]. Results of a 24-week oral NAC supplementation of cystic fibrosis patients revealed that NAC recipients maintained their lung function without a significant effect on the biomarkers of neutrophilic inflammation [56]. Another trial was prematurely terminated in 2018 due to the absence of between-group differences in the rates of contrast-associated acute kidney injury; there was no noticeable benefit of the oral NAC on the contrast-associated acute kidney injury prevention, no noticeable improvement on the need for dialysis, persistent kidney injury or death in subjects at high risk of renal complications because of angiography [57]. Similar conclusions were reached from the “Acetylcysteine for contrast-induced neuropathy” trial [58].

Pre-clinical studies imply that NAC could have more uses in supportive care and preventing human disease. Examples include Alzheimer’s disease [59,60], asthma [61], inflammatory bowel disease [62], influenza [63], intrauterine growth retardation [64], obesity and insulin resistance [65,66,67,68], ischemic cardiovascular disease [69,70], heavy metal toxicity [71,72], diabetic neuropathy [73], and age-related memory impairment [74]. Due to its capacity to break down biofilms and improve antibiotic permeability, it is promising as an adjuvant antimicrobial drug [75]. Several pre-clinical studies have also demonstrated that NAC supplementation leads to life extension and diminished effects of aging, in invertebrates [76,77,78,79] as well as mammals [80] and in human breast epithelial stem cells [81]. Such findings have yet to be replicated in humans. This is likely not solely due to NAC’s radical scavenging activity but also at least in part to telomerase activation and apoptosis inhibition [82], as is evidenced also by its capacity to delay oocyte aging [83]. However, antioxidants have the potential to either lengthen or shorten lifespan, depending on the dose and redox balance [84].

The role of NAC in the prevention and treatment of cancer is controversial, and it is discussed in more detail below. NAC has also attracted considerable attention as a sports supplement that can reduce muscle fatigue, improve athletic performance, and aid muscle recovery [85]. Although NAC is a well-known antioxidant and an old generic drug with several established clinical applications, more potential uses are still inadequately investigated. One of the main challenges of NAC as a medicine and a supplement is its broad range of effects and applications, far too few of which are well studied, in spite of a large effort in conducting preclinical and clinical trials.

## 4. NAC in Prevention and Complementary Treatment of Cancer

The role of antioxidants and reactive oxygen species (ROS) in cancer is controversial [86]. Epidemiological studies on synthetic antioxidants supplementation are inconclusive and contradictory mainly due to (1) anti vs. pro-oxidative properties of antioxidant and (2) antioxidant involvement in intracellular signaling and redox regulation, which modulate proliferation, apoptosis, and gene expression [87]. This is of particular significance during cell malignant transformation. Antioxidants in general are able to reduce the frequency of the malignant transformation by directly sequestrating ROS or by induction of cellular repair and adaptive stress responses that are important in preventing cancer initiation. For example, in experimental models of breast cancer, N-Acetylcysteine (NAC) reduced cancer aggressiveness, proliferation, and increased apoptosis of cancer cells [88,89]. By decreasing oxidative stress and inflammatory mediators, NAC interferes with intracellular metabolic processes by repressing glycolysis and increasing mitochondrial functioning [90,91]. On the other hand, antioxidant treatment may increase survival of cancer/precancer cells administered after malignant transformation [86]. The antioxidant supplementation in tumor-bearing mice was associated with accelerated cancer progression and increased metastasis in some preclinical studies [92,93]. The combination of N-acetylcysteine (NAC) and soluble vitamin E analog Trolox increased the migration and invasive properties of human malignant melanoma cells in an endogenous mouse model of malignant melanoma [92]. Similarly, N-acetylcysteine and vitamin E accelerated lung cancer progression in mice by reducing survival and increased tumor progression by disrupting the ROS-p53 axis [55].

In patients undergoing cancer therapy, antioxidant supplementation may alleviate unwanted radiation and chemotherapy-induced toxicity by quenching free radicals but also reduce the efficacy of chemo- and radiotherapy. This may increase (malignant and non-malignant) cell survival by altering cellular signal transduction pathways that regulate cell proliferation [94]. The reduction of ROS by antioxidants can lead to the survival of pre-initiated tumor cells, even in unnatural matrix environments [95]. Thus, NAC may have dichotomous effects with respect to tumorigenesis and NAC administration may differ depending on the stage of malignant transformation. By enhancing resistance to oxidative stress and decreased apoptosis during cancer promotion, progression, and treatment stages, NAC supplementation may not always be beneficial, since it may increase cancer cell survival in altered matrix environments by antioxidant restoration of adenosine triphosphate (ATP) generation [96]. Further clinical studies should be performed to address whether NAC administration ameliorates toxic side effects of radiation and chemotherapy with or without affecting the treatment efficacy.

## 5. NAC as a Dietary Supplement

Like many antioxidants, NAC has been very successful in the pharmaceutical, dietary supplement, and nutraceutical markets. In 2016 alone, Europe consumed approximately 3908.2 MT, USA approximately 3005.4 MT, and India approximately 1392.3 MT. The global market for NAC is expected to grow at a compound annual growth rate of about 22% over the next five years, from 490 million USD in 2017 [97]. Sellers of dietary supplements make a number of claims about the potential of NAC to protect against environmental toxins and pollutants, treat diverse conditions, extend lifespan, and even increase testosterone levels in men—in spite of limited scientific evidence. Little to no reliable information is available about the effects experienced by numerous users of NAC as a dietary supplement. The NAC-containing product with the most reviews on Amazon.com (100% NAC powder 1 kg, 905 reviews) has an average rating of 4.6 out of 5 stars [98]. Similar ratings can be seen for other popular NAC products. The 95 reviews on the website WebMD [99] convey similar impressions. None of this qualifies as scientific data, but we infer that NAC is popular as a dietary supplement.

## 6. NAC as a Sports Supplement, Effects in Skeletal Muscle

The performance of NAC as a sports supplement is discussed in detail in a recent meta-analysis by Rhodes and Brakhuis [85]. There is a great variability of study results also because of heterogenous methodologies. However, some studies have shown very significant athletic performance increases during repeated bouts of intermittent exercise (up to 50%) with NAC supplementation, particularly in athletes who have the capacity to generate more ROS in their muscles during exercise [100]. It also appears that the benefits of NAC are more significant when muscles are in a pre-fatigued state, and thus the produced ROS can exceed the buffering capacity of the endogenous antioxidant system. One of the major challenges of using NAC as a sports supplement is in the dosage and timing of administration, which are not standardized. For example, the daily dose of NAC in the studies included by Rhodes and Brakhuis varied from 1.2 to 20 g, and the supplementation period from 8 days to minutes before the performance. The heterogenous effects of NAC in various studies reflect the fact that there is a multifactorial optimum to the redox state of various tissues that is challenging to tackle, and either too much or too little of an antioxidant can lead to performance decrease and damage. According to the Rhodes and Brakhuis meta-analysis, larger doses of NAC (>5g) have an increased potential to cause side effects. Even though these side effects are generally mild and limited to gastrointestinal disturbances, they can hamper athletic performance and thus defy the purpose of supplementation. However, the evidence for these side effects is limited, and in several of the studies included in Rhodes and Brakhuis meta-analysis no side effects were reported in spite of the large doses.

## 7. NAC as an Anti-Aging Supplement, Effects on Degenerative Processes

NAC can potentially be effective in degenerative processes caused by aging, for instance, in neurodegenerative disorders, neuropathic pain, and stroke [101]. The present findings from animal studies support a neuroprotective role of NAC in controlling age-related neurological disorders [102]. For instance, NAC protects against Cd-induced neuronal apoptosis in mouse brain partially by inhibiting ROS-dependent activation of Akt/mTOR pathway. The findings highlight that NAC may be exploited for prevention and treatment of Cd-induced neurodegenerative diseases [103]. Animal model results support the possibility that NAC could be explored in clinical trials for amyotrophic lateral sclerosis disease [104], as well as Alzheimer’s disease [105] and mild cognitive impairment [106]. Further animal studies have shown that it delays age-associated memory impairment [74] and improves aging-related myocardial dysfunctions [70]. Since oxidative stress plays a prominent role in the modulation of neuropathic pain, NAC could be a potential candidate for its alleviation [107]. Furthermore, NAC could be used in endotoxemic states to prevent oxidative damage [108]. This warrants some caution, because NAC was associated with cardiac performance depression in a human trial [109]. NAC has a potential to improve immune function among the elderly [110]. A recent meta-analysis has also revealed a positive effect of NAC on human cognition, in healthy as well as mentally ill individuals [111]. NAC may be helpful in chronic fatigue syndrome [112]. Topical NAC may prevent UV-associated photoaging of the skin [7]. The synthesis of GSH is decreased in the elderly, which increases oxidative stress, itself a propagator of aging. This effect can be reversed with dietary supplementation [23]. Many medical conditions with beneficial role of NAC that are listed above are aging-associated. Based on these facts and the known molecular mechanisms of NAC as an antioxidant, we can hypothesize that it has potential as an anti-aging supplement. The dosage and timing of administration are even more of a concern here than in the case of sports supplementation, since ameliorating the effects of aging would require its long-term use. This would also raise the question of potential long-term side effects, which remains to be answered.

## 8. Summary and Conclusion, Future Perspectives

NAC is an established generic mucolytic and paracetamol poisoning antidote, but the list of conditions it can potentially improve has grown steadily over the years, and so has its popularity as a dietary supplement. In in vitro as well as in animal experiments it has exhibited potent antioxidant properties, which make NAC a powerful tool for diseases and states where ROS are the major cause of damage. However, modulating the redox state of cells, tissues, and organs is a delicate matter, and turning the dial too far in the antioxidant direction can cause more harm than good. Combined with heterogenous methodologies and a lack of standardization, the results of different studies are bound to conflict, which complicates the deduction of NAC’s effects. This is the major setback, since without determining the necessity, dosage, and timing of administration, optimal balancing of the redox scales is not possible. Developing and implementing technologies to measure the personalized levels of ROS and other oxidants and adjust the doses of antioxidants accordingly instead of using them blindly would provide an advantage, but also clinical and technological challenges. There is a wealth of unexploited information in the form of thousands of anonymous users of NAC as a dietary supplement. Since there is no regulation or documentation of this usage, we are missing out on potential information about the effects of NAC (and other antioxidants) in large numbers of people. Even though (especially oral) administration of NAC has been safe, the results of clinical trials for many conditions are still indecisive. Like other supplemented antioxidants, it may be harmful in the case of cancer or premalignancy, but there seem no other obstacles to studying NAC in many other conditions. More clinical trials of its use in neurodegenerative diseases, addiction, and mental health disorders are underway. This will provide much-needed information on NAC, and may be relevant to the supplement users. At the same time, it may help people suffering from chronic degenerative conditions.

## Figures and Tables

**Table 1:** Overview of Food and Drug Administration FDA-approved N-acetyl-cysteine (NAC) drugs and their indications.

Route	Administration	Strength	No.*	Medical Condition/ Therapy Type	Indication
Injectable	Intravenous	200 mg/mL (6 g/30 mL)	7	Poisoning/ antidote	Acetaminophen overdose reduction; Prevention of acute hepatic injury; Hepatic injury from repeated supratherapeutic ingestion.
Effervescent tablet	Oral	500 mg 2.5 g	1		
Solution	Oral	10% 20%	3	Bronchopulmonary disorders/ Adjuvant therapy	Abnormal, viscid, inspissated mucous secretions in chronic** and acute*** bronchopulmonary disease; Pulmonary complications of cystic fibrosis; Tracheostomy care; Pulmonary complications associated with surgery; Use during anesthesia; Post-traumatic chest conditions; Atelectasis due to mucous obstruction and diagnostic bronchial studies****.
Solution	Inhalation	10% 20%	3		

*: Number of drugs, currently on the market. **: Chronic bronchopulmonary disease: chronic emphysema, emphysema with bronchitis, chronic asthmatic bronchitis, tuberculosis, bronchiectasis, and primary amyloidosis of the lung. ***: Acute bronchopulmonary disease: pneumonia, bronchitis, and tracheobronchitis. ****: Diagnostic bronchial studies: bronchograms, bronchospirometry, and bronchial wedge catheterization.

**Table 2:** NAC clinical trials registered at ClinicalTrials.gov [48]. The number of studies is displayed according to the study status, tested medical conditions of currently active studies, tested medical conditions in completed studies, study phase and tested medical conditions of currently active studies, study phase, and tested medical conditions of completed studies.

**Status**	**Count**
Completed	159
Not yet recruiting	14
Active	54
Withdrawn/terminated/suspended	24
Unknown status	40
**Grand Total**	**291**
**Medical Conditions (Active Studies)**	**Count**
Addiction	12
Cancer/chemotherapy side effects	5
Cardiovascular diseases	5
Gastrointestinal diseases	4
Genetic disorders	1
Graft/stem cell complications/trauma	4
Infectious diseases	1
Metabolic diseases	1
Neuro/psychiatric disorders	12
Obstetrics	2
Poisoning antidote	1
Pulmonary diseases	1
Surgery complications/trauma	5
**Grand Total**	**54**
**Medical Conditions (Completed Studies)**	**Count**
Addiction	17
Blood disorders	4
Cancer/chemotherapy side effects	2
Cardiovascular diseases	10
Dermatologic disorders	2
Gastrointestinal diseases	15
Genetic disorders	1
Infectious diseases	3
Metabolic diseases	8
Muscle disorders	1
Neuro/psychiatric disorders	24
Obstetrics	11
Ophthalmological diseases	5
ORL	5
Other	4
Poisoning antidote	2
Pulmonary diseases	13
Renal disorders	31
Surgery complications/trauma	1
**Grand Total**	**159**
**Phase/Medical Conditions (Active Studies)**	**Count**
**Early Phase 1**	**5**
Addiction	2
Metabolic diseases	1
Neuro/psychiatric disorders	1
Pulmonary diseases	1
**Not Applicable**	**10**
Cardiovascular diseases	2
Gastrointestinal diseases	1
Graft/stem cell complications/trauma	1
Neuro/psychiatric disorders	4
Obstetrics	1
Surgery complications/trauma	1
**Phase 1**	**4**
Addiction	1
Cancer/chemotherapy side effects	1
Neuro/psychiatric disorders	1
Poisoning antidote	1
**Phase 1|Phase 2**	**3**
Cancer/chemotherapy side effects	2
Gastrointestinal diseases	1
**Phase 2**	**13**
Addiction	6
Cancer/chemotherapy side effects	1
Gastrointestinal diseases	1
Genetic disorders	1
Graft/stem cell complications/trauma	1
Infectious diseases	1
Neuro/psychiatric disorders	2
**Phase 2|Phase 3**	**3**
Addiction	1
Graft/stem cell complications/trauma	1
Obstetrics	1
**Phase 3**	**8**
Cancer/chemotherapy side effects	1
Cardiovascular diseases	3
Neuro/psychiatric disorders	2
Surgery complications/trauma	2
**Phase 4**	**8**
Addiction	2
Gastrointestinal diseases	1
Graft/stem cell complications/trauma	1
Neuro/psychiatric disorders	2
Surgery complications/trauma	2
**Grand Total**	**54**
**Phase/Medical Conditions (Completed Studies)**	**Count**
**Early Phase 1**	**3**
Addiction	1
Blood disorders	1
ORL	1
**Not Applicable**	**18**
Cardiovascular diseases	1
Gastrointestinal diseases	3
Metabolic diseases	3
Neuro/psychiatric disorders	2
Obstetrics	2
Pulmonary diseases	2
Renal disorders	5
**Phase 1**	**22**
Addiction	3
Cancer/chemotherapy side effects	1
Cardiovascular diseases	1
Gastrointestinal diseases	2
Neuro/psychiatric disorders	4
Ophthalmological diseases	4
ORL	1
Other	4
Pulmonary diseases	1
Renal disorders	1
**Phase 1|Phase 2**	**12**
Addiction	2
Blood disorders	1
Infectious diseases	1
Metabolic diseases	3
Neuro/psychiatric disorders	2
Obstetrics	2
Renal disorders	1
**Phase 2**	**47**
Addiction	8
Blood disorders	1
Cancer/chemotherapy side effects	1
Cardiovascular diseases	4
Dermatologic disorders	1
Gastrointestinal diseases	2
Genetic disorders	1
Infectious diseases	1
Metabolic diseases	1
Muscle disorders	1
Neuro/psychiatric disorders	14
Obstetrics	1
Ophthalmological diseases	1
ORL	1
Pulmonary diseases	2
Renal disorders	7
**Phase 2|Phase 3**	**8**
Gastrointestinal diseases	1
Obstetrics	1
ORL	1
Renal disorders	5
**Phase 3**	**20**
Addiction	2
Blood disorders	1
Cardiovascular diseases	2
Dermatologic disorders	1
Gastrointestinal diseases	4
Infectious diseases	1
Obstetrics	2
Pulmonary diseases	2
Renal disorders	5
**Phase 4**	**29**
Addiction	1
Cardiovascular diseases	2
Gastrointestinal diseases	3
Metabolic diseases	1
Neuro/psychiatric disorders	2
Obstetrics	3
ORL	1
Poisoning antidote	2
Pulmonary diseases	6
Renal disorders	7
Surgery complications/trauma	1
**Grand Total**	**159**

ORL: Otorhinolaryngology.

**Table 3:** Medical conditions investigated by withdrawn, terminated, and suspended studies listed by ClinicalTrials.gov [48]. Listed: number of trials listed at ClinicalTrials.gov [48]; Phase: study phase; N/A: not applicable.

Status/Medical Condition	Listed	Phase	Termination Reason
**SUSPENDED**	4		
**Autoimmune Disorders**			
Systemic Lupus Erythematosus	1	1|2	Short of funds
**Cardiovascular Diseases**			
Cardiovascular Disease|Renal Insufficiency, Acute|Cardiopulmonary Bypass	1	4	Opposite result
**Infectious Diseases**			
Hepatitis C	1	N/A	Short of funds
**Metabolic diseases**			
Insulin Resistance|Metabolic Syndrome	1	N/A	N/A
**TERMINATED/**	15		
**Addiction**			
Acetaminophen Overdose	1	3	Insufficient enrollment
Prevention of Hangover Using NAC	1	N/A	Insufficient enrollment
**Cancer/Chemotherapy Side Effects**			
Bone Marrow Suppression|Brain and Central Nervous System Tumors|Drug/Agent Toxicity by Tissue/Organ|Long-term Effects Secondary to Cancer Therapy in Children	1	1	N/A
Malignant Ovarian Endometrioid Tumor|Malignant Ovarian Serous Tumor|Recurrent Fallopian Tube Carcinoma|Recurrent Ovarian Carcinoma|Recurrent Primary Peritoneal Carcinoma	1	2	Slow accrual
**Gastrointestinal Diseases**			
Acute Liver Failure|Fulminant Hepatic Failure	1	4	Insufficient enrollment
Drug Induced Liver Injury	1	N/A	2 sepsis cases after steroid admin.
**Genetic disorders**			
Cystic Fibrosis	1	4	Insufficient enrollment
**Infectious Diseases**			
*Helicobacter pylori* Infection	1	1|2	Efficacy of eradication: 2 out of 31
**Metabolic diseases**			
Type 2 Diabetes Mellitus|Hypertension	1	4	N/A
**Neuro/Psychiatric Disorders**			
Borderline Personality Disorder|Self-Injurious Behavior	1	2	Poor subject compliance
Bulimia Nervosa	1	2|3; 3	No meaningful improvements
Obsessive-Compulsive Disorder	1	2	Insufficient enrollment
**Pulmonary Diseases**			
COPD|Chronic Bronchitis	1	N/A	PI’s discretion
**Renal Disorders**			
Chronic Kidney Failure	1	N/A	N/A
**Surgery Complications/Trauma**			
Ischemic Reperfusion Injury|Insufficiency; Hepatic, Postoperative|Liver Tumour	1	2	N/A
**WITHDRAWN/**	4		
**Cancer/Chemotherapy Side Effects**			
Ovarian Carcinoma, Stage 3 or 4|Epithelial Ovarian Carcinoma|Primary Peritoneal Carcinoma	1	1	No funding for the cost of NAC
**Gastrointestinal Diseases**			
Liver Failure|Liver Failure, Acute|Drug Induced Liver Injury|Prevention and Control|Fever	1	N/A	Short of funds
**Neuro/Psychiatric Disorders**			
Autistic Disorder|Seizures|Irritability	1	N/A	No eligible subjects located
Posttraumatic Stress Disorder	1	2	Cancelled research project
**Grand Total**	**23**		
